# Secondary consumption of mycorrhizal fungi by two endangered marsupial carnivores: the spotted-tailed quoll (*Dasyurus maculatus*) and Tasmanian devil (*Sarcophilus harrisii*)

**DOI:** 10.1007/s00442-026-05927-0

**Published:** 2026-07-10

**Authors:** Conor Nest, Timothy Henderson, Todd F. Elliott, Guy-Anthony Ballard, Karl Vernes

**Affiliations:** 1 Ecosystem Management, School of Environmental and Rural Sciences, University of New England, Armidale, NSW Australia; 2 Department of Biology, Duke University, Durham, NC USA; 3 Vertebrate Pest Research Unit, Department of Primary Industries, University of New England, Armidale, NSW Australia; 4 Natural History Museum, University of New England, Armidale, NSW Australia

**Keywords:** Dasyuridae, Fungal ecology, Mammal ecology, Mycophagy, Secondary dispersal

## Abstract

Mycorrhizal fungi form symbiotic relationships that are vital to nutrient and water acquisition by plants, with many mycorrhizal fungi requiring animal-mediated dispersal. Primary mycophagists often have relatively small home ranges, meaning fungal dispersal distances can be relatively short. However, fungal spores can be incidentally consumed and dispersed when a predator or scavenger eats mycophagous prey. These secondary consumers often move greater distances than their prey, which enables the long-distance dispersal (LDD) of fungal spores. In this study, we aimed to determine whether Australia’s two largest extant marsupial carnivores, the spotted-tailed quoll (*Dasyurus maculatus*) and Tasmanian devil (*Sarcophilus harrisii*), are acting as secondary dispersers of mycorrhizal fungi through the consumption of mycophagous prey. Quoll trapping and scat collection was undertaken at three sites in eastern New South Wales, whilst Tasmanian devil scats were collected opportunistically at three sites in Tasmania. Scats from these predators were analysed for the presence of fungal spores and prey animals. Quolls consumed 20 mammal species, including 14 that were identified as mycophagous. Across all three sites, 72.3% of quoll scats contained fungi, with a total of 77 fungal taxa identified. Quoll scats containing mycophagous mammals contained significantly more fungal taxa than those without mycophagous mammals present. Tasmanian devil scats contained six fungal taxa, with 33% of scats containing fungi, but a small sample size precluded further analysis. Our study indicates that spotted-tailed quolls and Tasmanian devils are both likely to be providing a previously unreported ecosystem service through the LDD of mycorrhizal fungi.

## Introduction

Mycorrhizal fungi colonise plants through the spread of their mycelial network or dispersal of their spores (Jonsson et al. 1999; Peay et al. 2012). These symbiotic relationships give plant roots improved access to water and soil nutrients (Allen and Allen 1991), can enhance plant drought tolerance (Rapparini and Peñuelas 2014), and increase disease resistance (Branzanti et al. 1999). In hypogeous (belowground) or sequestrate (enclosed) fungi, spores are produced inside the fruiting bodies and wind dispersal is unlikely to occur. Instead, animal-mediated dispersal is necessary, with fruiting bodies ingested and spores defecated back into the environment (Fogel and Trappe 1978). A mycophagous (fungus-feeding) animal’s contribution to dispersal of mycorrhizal fungi differs depending on the distance that they disperse fungal spores, as well as the quantity and diversity of the spores they ingest (Schickmann et al. 2012). Some species may disperse large quantities of spores over a short distance, maintaining local distributions of fungi, whilst others may disperse relatively few spores over long distances (long-distance dispersal; LDD), facilitating range expansions and gene exchange between fungal populations (O’Malley 2012).

However, fungal spores are not exclusively dispersed by mycophagous species, but can be dispersed incidentally by secondary consumers (Elliott et al. 2024; Stephens et al. 2025; Vogilino 1895). Secondary consumption occurs when a primary consumer of propagules (seeds and spores) is eaten by a predator which incidentally ingests propagules that the primary consumer ate (Hämäläinen et al. 2017). The predator then disperses and deposits the propagules in their scat or, in the case of owls, a regurgitated pellet (Dean and Milton 1988; Nogales et al. 2007, 2012). Secondary consumers are generally larger than primary consumers and typically have larger home ranges (Carbone et al. 2005; Swihart et al. 1988), meaning that deposition site and dispersal distance can vary dramatically between primary and secondary dispersers (Dean and Milton 1988; Nogales et al. 2007, 2012). LDD events, facilitated by secondary dispersers, likely assist in the recolonisation of disturbed, fragmented, or degraded habitats by mycorrhizal fungi (Bent et al. 2011; Claridge et al. 2001; Kipfer et al. 2011; Torrez et al. 2016), as well as vegetation shifts in response to climatic cycles or fire regimes (Vernes and Dunn 2009). LDD events are important for forest resilience, regeneration, and succession (Schickmann et al. 2012), making their importance disproportionate to their frequency (Nathan et al. 2008). Other ecological benefits of secondary dispersal include the deposition of propagules in previously uncolonised locations (Hämäläinen et al. 2017) and a potential increase in mycorrhiza formation due to longer gut retention times freeing more spores from the asci and degrading spore walls to a greater extent (Ori et al. 2021).

Secondary consumption of fungal spores is relatively poorly studied (Vašutová et al. 2019), but has been found in a range of taxa including centipedes and salamanders (Lilleskov and Bruns 2005), toads (Vogilino 1895), woodpeckers (Watson and Shaw 2018), kestrels (Watling 1963), and microbats (O’Malley 2012), as well as larger predatory mammals such as North American fishers (*Pekania pennanti*), red foxes (*Vulpes vulpes*), bobcats (*Lynx rufus*), coyotes (*Canis latrans*), wolves (*Canis lupus*) and dingoes (*Canis familiaris*) (Elliott et al. 2024; Schickmann et al. 2012; Stephens et al. 2025). Despite a lack of records, secondary consumption of fungi is thought to be common in both birds and reptiles, particularly snakes and predatory birds that feed on mycophagous mammals (Elliott et al. 2019a, b; Trappe and Claridge 2005). It is likely that many secondary consumers are dispersing fungi incidentally. The movement of spores through food webs has the potential to reach a higher trophic level than secondary consumption, namely when a secondary consumer is predated by a higher-order predator, but evidence of this is still lacking.

The spotted-tailed quoll (*Dasyurus maculatus*; hereafter referred to as ‘quoll’) and Tasmanian devil (*Sarcophilus harrisii*), are the two largest extant marsupial carnivores in Australia, weighing 1.4–6.1 kg (Green and Scarborough 1990) and 7–9 kg (Menkhorst and Knight 2011) respectively. Quolls occur along the east coast and adjacent ranges of mainland Australia and coexist with Tasmanian devils across Tasmania (Menkhorst and Knight 2011). The north Queensland and south-eastern mainland populations of quolls are currently listed as Endangered under the *Environment Protection and Biodiversity Conservation Act 1999* (EPBC Act) (Department of Climate Change, Energy, the Environment and Water [DCEEW] 2026), whilst the Tasmanian population is currently listed as Vulnerable. Threats to quolls include persecution by humans, roadkill, poisoning from invasive cane toads (*Rhinella marina*), habitat loss and fragmentation, predation by and competition with introduced predators, and climate change (Burnett and Marsh 2004; Rowland et al. 2023; Uzqueda et al. 2020). The Tasmanian devil is currently listed as Endangered (DCCEEW 2026), with devil facial tumour disease (DFTD) the primary threat to the species, causing a 77% decline in median density within five years of the disease’s emergence in 1996 (Lazenby et al. 2018) and a 68% total population decline by 2020 (Cunningham et al. 2021). Other threats to Tasmanian devils include human persecution, roadkill, potential competition with feral cats, dog attacks and habitat fragmentation (Jones 2000; Lawrence and Wiersma 2019).

Quolls and Tasmanian devils both feed on a wide range of mammals, including other dasyurids such as *Antechinus* sp., peramelids, phalangerids, pseudocheirids, potoroids, macropods and murids, as well as reptiles, birds, frogs and invertebrates (Andersen et al. 2017a; Belcher 1995; Dawson et al. 2007; Jarman et al. 2007; Pemberton et al. 2008). Given the large number of mycophagous species present in south-eastern Australia (Elliott et al. 2022, 2023; Nest et al. 2023, 2025; Nuske et al. 2017), including many that appear in the diets of quolls and Tasmanian devils, it is likely that both predators are consuming and dispersing fungi secondarily. Quolls can travel up to 12.5 km in a single night (Hamer et al. 2021), whilst Tasmanian devils have been recorded moving up to 21.7 km in a single night (Thalmann et al. 2015), meaning that both species likely facilitate significant LDD of mycorrhizal fungi. In this study, we aim to determine if quolls and Tasmanian devils are acting as secondary dispersers of mycorrhizal fungi in south-eastern Australia. We also examine whether the secondary consumption of mycorrhizal fungi by quolls differs between three sites in New South Wales (NSW), Australia and how the consumption of mycophagous mammalian prey relates to the presence of mycorrhizal fungi in quoll scats.

## Methods

### Study area

We conducted quoll trapping across three sites in NSW; Mt Royal National Park (MRNP; 32°10’56"S, 151°18’56"E), a biodiversity offset area (hereafter referred to as “Hebden”; 32°19’22"S, 151°00’23"E) in the Hunter Valley region, and Cathedral Rock National Park (CRNP; 30°26’40"S, 152°14’52"E). Tasmanian devil scats were collected opportunistically from three locations in Tasmania: Narawntapu National Park (41°6’3"S, 146°38’48"E), Franklin-Gordon Wild Rivers National Park (42°12’11"S, 145°57’51"E) and a private property near The Lea (42°56′08″S, 147°18′56″E), approximately 6 km south of Hobart.

MRNP contains a variety of intact forest types and vegetation communities, including cool temperate rainforest, tall open forest and wet sclerophyll forest (NSW National Parks and Wildlife Service [NPWS] 2010). The terrain is steep and mountainous, with Mt Royal rising to 1586 m asl.

Hebden is located approximately 20 km south-west of MRNP and 3 km from an open cut coal mine. It consists of a mix of severely fragmented habitat that is degraded from agriculture and mining, and remnant areas of grassy woodlands, sclerophyll forests and pockets of dry rainforest (Henderson et al. 2021). Approximately one third of the site consists of forest fragments, and the terrain is steep, with many rocky outcrops and gullies (Henderson et al. 2021).

CRNP has an undulating terrain interspersed with rugged granite outcrops and highland swamps, with altitude varying from 1,100 m to 1,584 m asl. Much of the park is covered in dry sclerophyll forest, with small areas of rainforest in the wetter gullies (NSW NPWS 2002). The park lacks fast flowing streams, allowing areas of subalpine fen, fen-heath and mallee heath plant communities (NSW NPWS 2002).

Narawntapu National Park is located on the central-north coast of Tasmania and contains a variety of habitats, including grasslands, ex-agricultural pastures, dry sclerophyll woodlands, bracken/scrubland and coastal heathland (Martin et al. 2018). Franklin-Gordon Wild Rivers National Park is in the central-west of Tasmania and consists of a mix of temperate rainforest, wet sclerophyll forest, subalpine woodlands and button grass moorlands. The private property near The Lea is dominated by dry sclerophyll woodland and forest, interspersed with open pasture (Elliott et al. 2025).

## Field methods

Quoll trapping was undertaken between June 2019 and August 2021, with sampling predominately occurring between May and August. Trapping sessions varied from 2–7 nights at a time, with 20–66 cage traps (60 × 34 × 34 cm, Mascot Wire Works, Enfield South, NSW) deployed at a time. Traps were covered with plastic sheeting, baited with chicken thighs and deployed adjacent to tracks and trails. Traps were generally deployed at 500 m intervals, but were occasionally placed closer together in good habitat (e.g. wooded creek lines in fragmented habitat). Traps were set in the late afternoon and checked in the early morning, with captured animals transferred into a triangular canvas bag for weighing, measuring and other data collection unrelated to this study (Henderson 2023). Once processed, the animals were given a general health check and released into cover. Scats were collected from the handling bags and the cage traps, stored in plastic ‘zip-lock’ sandwich bags and frozen within 12 hours.

Quoll scats were also collected opportunistically when found, often from latrine sites, with hair analysis undertaken to confirm scat identification. Latrine sites are usually rocky outcrops or creek lines where several quolls may come to defecate (Belcher 1994; Kruuk and Jarman 1995). To ensure independence of sampling, only a single scat from each latrine site was included in the analyses. Similarly, if an individual quoll was trapped on consecutive nights, all scats collected subsequent to the first night were disregarded. However, if there was at least one night between captures on a trapping session, the samples were included, as the individual was likely to have fed naturally between captures.

Tasmanian devil scats were collected opportunistically from the ground whilst conducting other mammal fieldwork in Aug-Sep 2019. Scat were stored in plastic ‘zip-lock’ sandwich bags and frozen within 12 hours. Tasmanian devil scats were identified based on size, shape, colour, odour and the presence, size and state of digestion of bone fragments (Andersen et al. 2017a). Tasmanian devil scats can be differentiated from other carnivore scats by their large size and tightly twisted and cylindrical shape (Triggs 1996). As Tasmanian devils consume and digest large amounts of bone, their scats have a grey tinge and frequently contain sizeable bone shards (Andersen et al. 2017a). Only six Tasmanian devil scats were collected, four from Narawntapu NP, one from Franklin-Gordon Wild Rivers NP and one from private property near the Lea. The four scats from Narawntapu NP were collected within a 2 km area, but no attempt was made to ensure independence of sampling. This was due to the small number of Tasmanian devil scats available for faecal analysis, which precluded any statistical analysis.

## Preparation of faecal samples

Subsamples of each scat collection were taken, comprising approximately 25 g of scat taken from multiple sections of the whole scat (for example: two end pieces and a middle piece). Subsamples of each scat collection were soaked in 70% ethanol, macerated, then rinsed through a 125 × 125 μm mesh with distilled water. The filtrate was then centrifuged using an ELMI CM-7 S for three minutes at 3000 rpm. Excess liquid was decanted, and six to eight drops of resultant filtrate were placed on a glass slide and left to dry. The sample was then mounted in glycerine jelly and sealed with a glass coverslip. Slides were systematically scanned under an Olympus CX41 light microscope at 400x magnification, with 10 random fields of view examined for fungal spores per slide. Morphological characteristics such as size, shape, ornamentation, symmetry and wall-thickness were used to identify spore types to the lowest taxonomic level possible. Spore mounts from fruiting body collections made by the authors and by Danks et al. (2013) in the same or similar habitats were used to aid in spore identification. Where we could reliably distinguish different spore types from within the same genus or family, identification numbers were assigned (e.g. *Cortinarius* 1). Identification numbers used in this study are not necessarily sequential, as they relate to other taxa in the region that have been identified in related studies (C. Nest, unpubl. data). Several spore types could not be reliably identified, but were distinctive enough to be classed as a separate dietary fungal taxon and were nominated as ‘Unknown 1’, ‘Unknown 2’ etc. Coprophilous and micro fungi were outside the scope of this study; we focused solely on presence and diversity of mycorrhizal taxa.

Any quoll scat remaining after taking subsamples for microscopic analysis was dried in an oven for 24 h at 100 degrees Celsius and sent to ScatsAbout (http://www.scatsabout.com.au) for analysis, where a reference hair collection and macroscopic/microscopic characteristics of hair and bone were used to identify prey animals (Brunner and Coman 1974). Grooming hairs were also used to confirm the identity of the predator in any of the scats that were collected opportunistically. Several of the scats that were collected from trapped quolls and analysed for fungal spore presence were not sent for hair analysis, as they were of insufficient size for effective hair analysis.

## Data analyses

Statistical analyses were performed in R v4.5.0 (R Core Development Team 2025). Differences in the number of fungal taxa present in the scats from each site were examined using an Analysis of Variance (ANOVA) test. Multivariate analyses available in the R package vegan (Oksanen et al. 2019) were then used to examine between-site trends in the diversity of fungal taxa present in the quoll scats. The ‘outlier’ function was used to identify extreme outliers in the data (Wildi 2017), though none were identified. Pairwise permutational multivariate analysis of variance (PERMANOVA) was performed using the ‘adonis2’ function to identify any differences in the fungal taxa consumed between sites. Since this test is sensitive to data dispersion and may therefore confuse within-group variation with among-group variation (Anderson 2001), we performed an analysis of multivariate homogeneity with the ‘betadisper’ function, followed by ANOVAs to test if groups differed in their dispersion. Tukey HSD *post hoc* tests were then undertaken to examine if and which groups differ in relation to their variances.

A Generalised Linear Mixed Model (GLMM) was used to determine whether the number of mycophagous prey mammals (fixed effect) in a quoll scat predicted the presence of fungal spores (response variable), with site included as a random effect. We used binomial distribution because residual diagnostics indicated that there was no overdispersion or zero-inflation. Mycophagous prey mammals were identified from published literature as any species that has been recorded consuming fungi (Elliott et al. 2022). A GLMM was also used to examine whether the presence of mycophagous prey mammals (fixed effect) in a quoll scat significantly increased the number of fungal taxa present (response variable), with site included as a random effect. For this model, Poisson distribution showed significant overdispersion, and was therefore re-fitted with negative binomial distributions. Although there is evidence of mycophagy among various birds and reptiles in eastern Australia (Cooper and Vernes 2011; Elliott and Elliott 2019; Elliott and Vernes 2019, 2021) we did include them in this analysis due to lack of clarity on which species were consumed and levels of mycophagy.

Further analysis was undertaken by partitioning prey mammal species into small (< 500 g), medium (500 g–10 kg) and large (> 10 kg) size classes, based on published mean body weights (Menkhorst and Knight 2011). The size designations for each prey mammal species are displayed in Table 1. Scats that contained multiple species from more than one size class were excluded. A GLMM was used to determine whether mammalian prey size class (fixed effect), significantly influenced the number of fungal taxa present (response variable), with site included as a random effect. For this model, Poisson distribution showed significant overdispersion, and was therefore re-fitted with negative binomial distributions. We assessed the assumptions for the models by evaluating the model fit and residual patterns using the ‘simulateResiduals’ function from the DHARMa package v.0.4.7 (Hartig 2026). The models were fitted using the ‘glmer’ or ‘glmer.nb’ function for the lme4 package (Bates et al. 2015). We used a blocked PERMANOVA to test whether the fungal taxa present in the quoll scats differed between prey size classes. Site (n = 3) was used as the blocking factor, with Bray-Curtis dissimilarities as the distance measure.

**Table 1:** A list of 25 prey taxa identified in quoll scats from MRNP, Hebden and CRNP. Numbers represent the number of scats that contained the given taxon. N = the number of scats analysed for prey species. Bolded prey species are recognised in the literature as being mycophagous to some degree (Elliott et al. 2019a, 2022). Hair analysis data presented from Hebden is also presented in Henderson and Nest (2024)

Prey taxa		Prey size	MRNP	Hebden	CRNP	Total
			*N = 71*	*N* = 20	*N* = 22	*N* = 113
Family	Species					
Tachyglossidae	*Tachyglossus aculeatus*	Medium	2			2
Dasyuridae	***Antechinus stuartii***	Small	1			1
Peramelidae	***Isodon macrourus***	Medium		3		3
Phalangerdidae	***Trichosurus caninus***	Medium	3	2	1	6
	***Trichosurus vulpecula***	Medium	1	1		2
Petauridae	*Petaurus norfolcensis*	Small	2		1	3
Pseudocheiridae	*Petauroides volans*	Medium			1	1
	*Pseudocheirus peregrinus*	Medium	6	1	2	9
Macropodidae	***Notamacropus parma***	Medium	2			2
	***Notamacropus rufogriseus***	Medium		1	1	2
	***Thylogale*** **sp.**	Medium	6			6
	***Thylogale stigmatica***	Medium	17		1	18
	***Thylogale thetis***	Medium	6		4	10
	***Wallabia bicolor***	Large	8		6	14
Pteropodidae	*Pteropus poliocephalus*	Medium	1	1	1	3
Muridae	***Mastacomys fiscus***	Small		1		1
	***Mus musculus***	Small		4	4	8
	***Rattus fuscipes***	Small	5			5
	***Rattus lutreolus***	Small	4			4
	***Rattus rattus***	Small	4	7	1	12
Leporidae	Hare/Rabbit	Medium		1	2	3
Scincidae	***Tiliqua scincoides***		1		1	2
	Skink sp.		1	3		4
Unknown Family						
	Bird		8		1	9
	Frog		1			1
	Invertebrate		2	4	2	8
	Plant		3	1		4

To disentangle the effects of differing sample sizes on the total number of fungal taxa consumed by each species, species accumulation curves were developed using the ‘specaccum’ function. The function ‘specpool’ was then used to calculate several estimates for total fungal taxa richness (Chao 2, Jackknife 1, Jackknife 2, Bootstrap), in the diet of each mammal species.

## Results

### General trends

Across the three sites, 72.3% of quoll scats contained fungal spores, with 77 fungal taxa identified. Scats from CRNP (*n* = 22 scats analysed for fungi and prey species) contained 12 mammalian prey species and evidence of 18 fungal taxa (Tables 1 and 2). Scats from MRNP (*n* = 72 scats analysed for fungi, *n* = 71 scats analysed for prey species) contained 14 mammalian prey species and evidence of 64 fungal taxa (Tables 1 and 2). Scat from Hebden (*n* = 25 scats analysed for fungi, *n* = 20 scats analysed for prey species) contained 10 mammalian prey species and evidence of 28 fungal taxa (Tables 1 and 2). There were only three quoll scats that contained more than 10 fungal taxa, these were all from MRNP. The greatest number of fungal taxa found in a single scat was 19, with swamp wallaby (*W. bicolor*) the only prey species recorded in that scat. Two of the six Tasmanian devil scats that were analysed contained fungi, with six fungal taxa recorded in total. These fungal taxa were Boletoid 4, *Cortinarius* 1, *Cortinarius* 10, *Mesophellia*, *Rossbeevera* 1 and Unknown 11. The small sample size precluded any statistical analysis, but still provides the first evidence of Tasmanian devils as mycorrhizal spore dispersers.

**Table 2:** A list of 77 fungal taxa identified in quoll scats from MRNP, Hebden and CRNP. Numbers represent the number of scats that contained the given taxon. N = the number of scats analysed for fungal spores

Fungal taxa	MRNP	Hebden	CRNP	Total
	*N* = 51	*N* = 19	*N* = 16	*N* = 86
*Amylascus*	1			1
*Andebbia*	2	1		3
*Aroramyces* 1	1		2	3
*Aroramyces* 2	2		1	3
*Aroramyces* 3	1			1
*Austrogautieria* 1	1			1
Bolbitaceae 2	1			1
*Boletellus*	1			1
Boletoid 1	1		1	2
Boletoid 2	15	7	14	36
Boletoid 3			1	1
Boletoid 4		1		1
Boletoid 5		1		1
*Cortinarius* 1	7			7
*Cortinarius* 2	4		1	5
*Cortinarius* 4	3	1		4
*Cortinarius* 6			2	2
*Cortinarius* 7	2	2		4
*Cortinarius* 8	14	7	4	25
*Cortinarius* 9	2	1		3
*Cortinarius* 10	4		2	6
*Descolea* 2	1			1
*Descolea* 3	3	1		4
*Descolea* 4	2			2
*Descolea* 6	9	2		11
*Descolea* 8	2			2
*Descolea* 12	1			1
*Descolea* 14		1		1
*Descolea* 15	8			8
*Descolea* 17	1	1		2
*Descolea* 19		1		1
*Descolea* 20	1			1
*‘Discina-like’* 2	1	1		2
*Elaphomyces* 1	3	2		5
*Elaphomyces* 3			1	1
*Elaphomyces* 4	1			1
*Gautieria*	1			1
*Glomus* 2	1		1	2
*Glomus* 3	2			2
*Glomus* 5	1			1
*Hydnangium*	15	8	1	24
*Hymenogaster* 1	1			1
*Labyrinthomyces* 1	1	1		2
*Labyrinthomyces* 2	1			1
*Mesophellia*			3	3
*Pisolithus*	1			1
*Rossbeevera* 1	3		1	4
*Rossbeevera* 2			1	1
*Rossbeevera* 3	3	1		4
Russulaceae 1	1			1
Russulaceae 2	3	2		5
Russulaceae 3	4	1		5
Russulaceae 6	1	1		2
Russulaceae 7	10	1	1	12
Russulaceae 8	1			1
Russulaceae 9		2		2
Russulaceae 10	2	2		4
Russulaceae 12	4			4
*Scleroderma* 1	1			1
*Scleroderma* 2	5		1	6
*Scleroderma* 3	5			5
*Scleroderma* 4			1	1
*Scleroderma* 5	1			1
*Scleroderma* 6	2			2
*Sclerogaster*	3			3
Unknown 1	2	2		4
Unknown 2	2			2
Unknown 3	2			2
Unknown 4	1			1
Unknown 6	1			1
Unknown 7	1			1
Unknown 9		1		1
Unknown 10	1			1
Unknown 11		1		1
Unknown 12	1	1		2
Unknown 13	1			1
Unknown 16	5			5
Total spore taxa	64	28	18	77

## Between-site differences in fungal presence

**Fig. 1:**
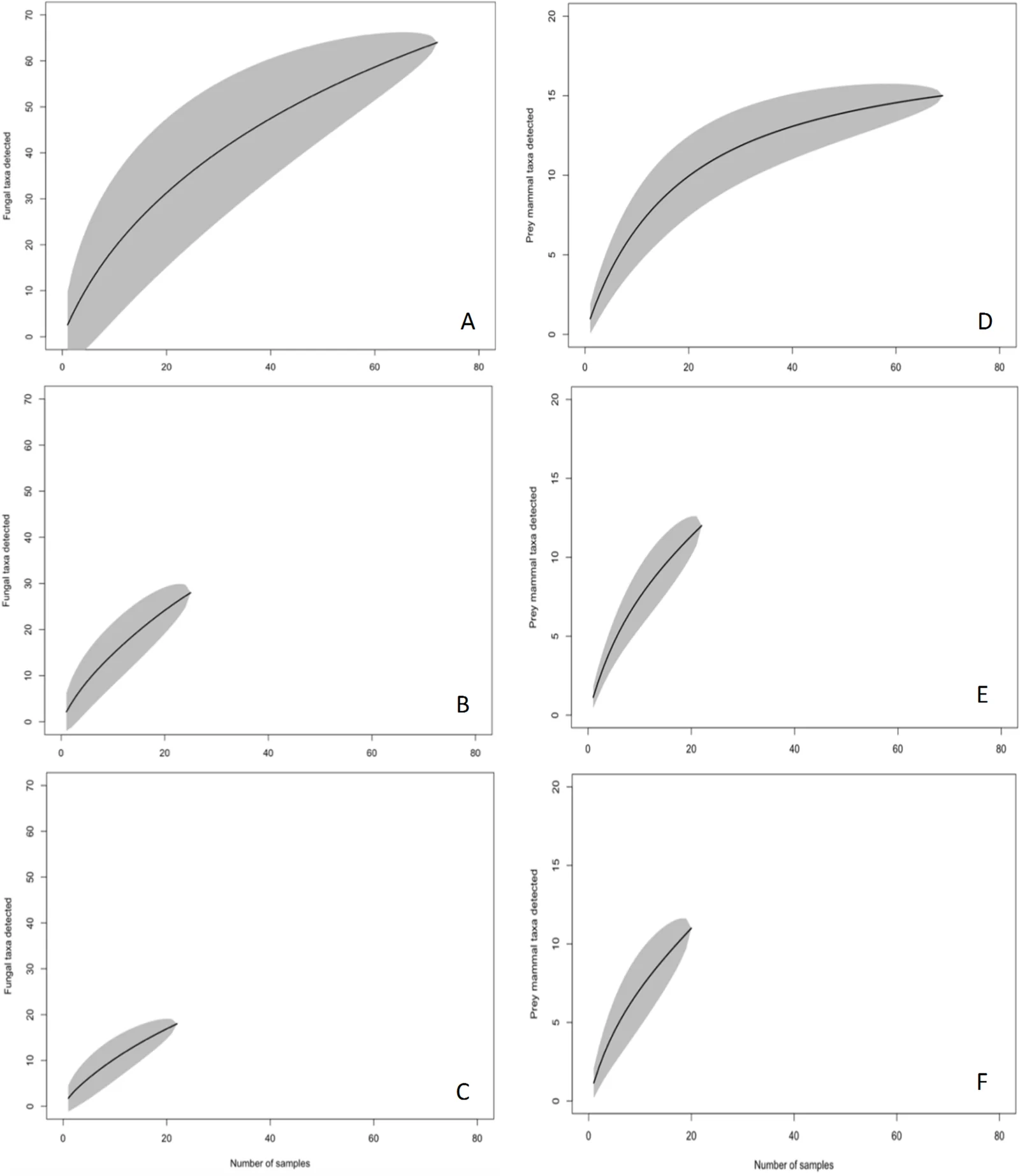
Species accumulation curves of the number of fungal taxa consumed by quolls from MRNP (**A**), Hebden (**B**) and CRNP (**C**), and the number of prey mammal taxa consumed by quolls at MRNP (**D**), Hebden (**E**) and CRNP (**F**)

There were no significant between-site differences in the mean number of fungal taxa present in the quoll scats (F_2, 108_ = 0.721, *p* = 0.489), whilst the proportion of scats containing fungi was relatively similar across all sites. PERMANOVA analysis found that site had a significant effect on the fungal taxa present (R^2^ = 0.088, F_2,83_ = 3.944, *p* < 0.001), with CRNP significantly different from both MRNP (R^2^ = 0.091, F_1,65_ = 6.471, *p* < 0.001) and Hebden (R^2^ = 0.138, F_1,33_ = 5.265, *p* < 0.001). Hebden and MRNP were not significantly different to each other (R^2^ = 0.019, F_1,68_ = 1.289, *p* = 0.185). However, the dispersion of dietary fungal taxa differed significantly between sites (F_2,83_= 19.131, *p* < 0.001). MRNP and Hebden did not have significantly different dispersions (95% CI = -0.05 to 0.13, *p* = 0.531), but CRNP was significantly different to both MRNP (95% CI = 0.15 to 0.34, *p* < 0.001) and Hebden (95% CI = 0.09 to 0.32, *p* < 0.001). These results suggest that the significant PERMANOVA result was caused by the greater dispersion in the fungal diets of quolls from CRNP, compared to those from MRNP or Hebden. Species accumulation curves for all three sites are steep, indicating that sample sizes were insufficient to capture all the fungal taxa present in the diets of quolls at each site (Fig. 1). Different estimators of total fungal taxa richness at each site indicated that quolls at MRNP consume the greatest number of fungal taxa and quolls at CRNP the fewest (Table 3).

**Table 3:** Estimators of total dietary fungi taxon richness and total mammalian prey richness (Chao 2, Jackknife 1, Jackknife 2, Bootstrap) for quolls at MRNP, Hebden and CRNP

Site	Number of fungal taxa	Chao (s.e.)	Jack1 (s.e)	Jack2	Bootstrap (s.e)
MRNP	64	100.98 (18.26)	93.58 (8.04)	111.26	76.99 (5.32)
Hebden	28	50.22 (14.23)	45.28 (6.88)	55.67	35.36 (3.38)
CRNP	18	40.91 (19.31)	29.45 (4.31)	37.77	22.73 (2.20)
	Number of prey mammal taxa				
MRNP	15	16.48 (2.26)	17.96 (1.71)	18.00	16.58 (1.04)
Hebden	11	34.28 (29.58)	17.65 (2.51)	23.10	13.68 (1.26)
CRNP	12	23.69 (12.58)	18.68 (2.53)	23.31	14.79 (1.21)

## Dietary species

Hair analysis found 20 mammal species present in the diets of quolls across the three sites, in addition to eastern blue-tongues (*Tiliqua scincoides*), unidentified skinks, birds, frogs, invertebrates and plants. The species accumulation curve for MRNP flattens as sample size increases, indicating that the curve is approaching its asymptote and that the sample size for MRNP was large enough to capture most of the mammalian prey species present in the diet of quolls (Fig. 1). In contrast, the species accumulation curves for Hebden and CRNP are still steep, indicating that sample sizes were insufficient to capture all the mammalian prey species in the diets of quolls at these sites (Fig. 1). Different estimators of total mammalian prey richness indicted that quolls likely consume a similar diversity of mammalian prey at each site (Table 3).

Of the mammalian prey species, 14 are known to be mycophagous (Table 1). There was no significant relationship between the number of mycophagous mammal species present in a quoll scat and the presence of fungal spores (GLM: z = 1.572, *p* = 0.116) or between the number of non-mycophagous mammal species present in a quoll scat and the presence of fungal spores (GLM: z = -0.840, *p* = 0.401). However, scats with a mycophagous species present contained significantly more fungal taxa than those that did not contain a mycophagous species (GLM: z = -2.767, *p* = 0.006).

### Differences in fungal presence between mammalian prey size classes

There were no significant differences in the mean number of fungal taxa present in quoll scats between any of the mammalian prey size classes (GLM: *p* > 0.05). Twelve quoll scats contained large prey species, with 83% of these scats containing fungi. Medium-sized prey species appeared in 56 quoll scats, with 71% of these scats containing fungi. Small prey species appeared in 27 quoll scats, with 74% of these scats containing fungi. PERMANOVA analysis showed that prey size class had no effect on the fungal taxa present in the quoll scats (R^2^ = 0.023, F_2, 67_ = 0.802, *p* = 0.743).

## Discussion

### General trends in secondary consumption

This study is one of the first to directly examine the secondary consumption and dispersal of fungal spores by predators. Given the high proportion of quoll scats containing fungi and the diversity of fungal taxa present, quolls are clearly playing a role in the dispersal of mycorrhizal fungi. Tasmanian devils are also secondarily consuming fungi and are likely playing an important role in fungal dispersal, but our conclusions are limited due to a small sample size. Quoll diets across the three sites were dominated by mammals (Table 1), with a mix of terrestrial and arboreal species, a result common to other dietary studies in eastern Australia (Belcher 1995; Glen and Dickman 2006).

Whilst the presence of mycophagous mammals in the diet of quolls was not a significant predictor of the presence of fungal spores, scats that contained mycophagous mammals had a significantly higher mean number of fungal taxa present than those without mycophagists present. Similar analysis was undertaken by Elliott et al. (2024), where the presence of mycophagous mammals in dingo scats was a significant predictor of the occurrence of fungal spores. The non-significance of mycophagous mammals predicting the presence of fungal spores in the diet of quolls may be due to a range of confounding factors. Firstly, quolls in this study did not consume any specialist mycophagists (species for which fungi are their primary food source), instead consuming a range of fungal generalists (species that consume a range of food items, including fungi), which are likely consuming fungi to varying degrees. We observed several samples that contained the remains of a mycophagous species, but did not contain any fungal spores, indicating that these fungal generalists were not consuming fungi regularly enough to always have fungal spores present in their scats.

Secondly, we observed several samples that contained only non-mycophagous mammals or non-mammalian prey (invertebrates, birds, frogs, reptiles), which did contain fungal spores. This may be due to the non-mammalian prey consuming fungi (Elliott et al. 2019a, b), fungal spores being retained in the prey digestive tract for longer than large particulates like hair and bones (Danks 2012; Stephens et al. 2025), or quolls consuming fungi primarily. There is no published evidence of quolls (or Tasmanian devils) consuming fungi primarily, but other similarly sized omnivorous predators may do so (Stephens et al. 2025). When mycophagous mammal remains were present in quoll scats, significantly more fungal taxa were present than in scats where they were absent, indicating that spores are primarily from consuming prey.

There were a mix of hypogeous and epigeous fungi recorded in the diets of quolls and Tasmanian devils, with hypogeous taxa including but not limited to *Aroramyces*, *Austrogautieria*, *Elaphomyces*, *Hydnangium*, *Hymenogaster*, *Mesophellia* and *Rossbeevera*. Frequently recorded taxa such as *Cortinarius*, *Descolea* and Russulaceae contain both hypogeous and epigeous fungi, with several of the *Cortinarius* taxa we recorded having characters indicating that they were likely hypogeous. For other taxa within these mixed genera and families, we have no evidence to indicate their fruiting habits. Regardless, the presence of hypogeal fungal taxa in the diets of quolls and Tasmanian devils indicates the deliberate excavation and consumption of sporocarps, either by the predators or their mycophagous prey.

### Between-site differences in fungal presence

There were no between-site differences in the mean number of fungal taxa present in each quoll scat and no detectable between-site differences in the fungal diets of quolls, due to significantly greater dispersion in samples from CRNP than in samples from MRNP or Hebden. Species accumulation curves indicated that sample sizes were insufficient to capture the entire fungal diet of quolls at each site. Greater sample sizes for each site would be beneficial to capturing more of the fungal diets of quolls and more equal sample sizes between sites would potentially reduce the data dispersion.

Quolls from MRNP were predicted to consume the greatest number of fungal taxa, followed by quolls from Hebden, with quolls from CRNP predicted to consume the fewest fungal taxa. Given MRNP is an intact forest community, spanning several habitat types including rainforest, wet sclerophyll, and open woodland, it is unsurprising that a high diversity of fungi is present. Hebden is fragmented, with patches of dry sclerophyll, grassy woodland and dry rainforest interspersed with open agricultural paddocks, meaning a lower diversity of fungi than the intact forests of MRNP is expected. CRNP is largely dominated by intact dry sclerophyll forest, making it more homogenous than MRNP, but fungal diversity would be expected to be greater than the fragmented habitat of Hebden. A relatively smaller sample size for both CRNP and Hebden may have caused underestimates of true richness, but it is likely that MRNP has the most diverse fungal community, given the range of intact forest types it contains.

### Differences in fungal presence between mammalian prey size classes

The size of the mammalian prey species present in the quoll scats had no effect on the mean number of fungal taxa or which fungal taxa were present in the scat. The frequency of occurrence of fungi in quoll scats was also similar across the three size classes. Small prey species such as bush rats (*Rattus fuscipes*) are likely consumed entirely by a quoll when captured, but medium-sized prey such as pademelons or scavenged large prey such as swamp wallabies (*Wallabia bicolor*) are unlikely to be consumed entirely in a single sitting. If quolls preferentially avoided the stomach contents of these larger prey species, the secondary dispersal of fungal spores would be limited. However, given that prey size class had no effect on fungal presence, our results suggest that even whilst scavenging for large prey quolls consume at least some of the stomach contents incidentally. Additionally, previous research that deployed remote monitoring cameras opportunistically at carcasses found that quolls regularly feed on the stomach of their prey (T. Henderson, unpubl. data). Similarly, Tasmanian devils often consume up to 90% of large, scavenged carcasses, including bones, organs, intestines and the contents of the intestines (Pemberton 1990), meaning they are also likely to secondarily consume at least some fungal spores regardless of the size of their mycophagous prey.

### Comparisons with other secondary consumers of fungi

There have been two other studies of secondary dispersal in eastern Australia, one on insectivorous microbats (O’Malley 2012), the other on dingoes (Elliott et al. 2024). The scats of eight microbat species from the New England Tablelands were found to contain fungal spores, with 49% of scats containing 23 fungal taxa. Microbats that consume mycophagous insects are likely to disperse fungal spores several to tens of kilometres given their capability of flight and gut retention times of up to 25 h (Morris et al. 1994; O’Malley 2012). Secondary consumption of fungi by dingoes was also examined on the New England Tablelands, where 28% of scats contained 14 fungal taxa (Elliott et al. 2024). Dingoes have been predicted to regularly disperse fungal spores at least two kilometres from the point of ingestion, occasionally dispersing spores up to 10.7 km (Elliott et al. 2024). In North America, five mammalian predators have been recorded consuming fungi secondarily; fishers (77% of scats), red foxes (84% of scats), bobcats (62% of scats), coyotes (65% of scats) and grey wolves (49% of scats) (Stephens et al. 2025). All species were predicted to disperse fungal spores over a kilometre from the point of ingestion, with grey wolves predicted to disperse some spores over four kilometres (Stephens et al. 2025).

Quolls can travel up to 12.5 km in a single night (Hamer et al. 2021), whilst Tasmanian devils have been recorded moving up to 21.7 km in a single night (Thalmann et al. 2015), but there is no published data on gut-retention times for either species. Given gut retention times generally increase with body size (Draper et al. 2022; Hume 2003), spore dispersal distances for quolls and Tasmanian devils would be expected to be less than those for dingoes or grey wolves and potentially similar to red foxes or fishers, which disperse spores a mean distance of 800 m and 2000 m respectively (Stephens et al. 2025).

Both quolls and Tasmanian devils are well established users of latrine sites, which are important for scent marking and communication between animals, particularly in the breeding season (Belcher 1994; Pemberton 1990; Ruibal et al. 2011). Both species are therefore unlikely to distribute fungal spores evenly across a landscape, instead concentrating them around latrine sites. The degree to which they use the latrine sites may also change throughout the year. However, both species also defecate randomly across the landscape and have several latrine sites that they may visit (Kruuk and Jarman 1995; Shier et al. 2023).

The western quoll (*D. geoffroii*) has previously been identified as a fungal consumer, consuming 18 spore types, but it is unclear whether primary or secondary consumption was occurring (Gaskin 2002). Similar to their larger dasyurid relatives, western quolls feed on a variety of species, including possums, rodents, bandicoots, macropods, other dasyurids, microbats, reptiles and birds (Glen et al. 2010; Moseby et al. 2022; Soderquist and Serena 1994; West et al. 2019). However, invertebrates and plant material are more prevalent in the diet of western quolls, with invertebrates occurring in up to 84% of scats (Soderquist and Serena 1994), and plant material in up to 30% (Moseby et al. 2022). Given the presence of mycophagous species in the diet of western quolls, it is likely that at least some secondary mycophagy is occurring, but the greater prevalence of plant material than in spotted-tailed quoll or Tasmanian devil diets may indicate a greater likelihood of some primary mycophagy occurring too. Interestingly, the presence of microbats in the diet of western quolls (Moseby et al. 2022), which are known secondary mycophagists (O’Malley 2012), illustrates the potential for fungal spores to travel through a third trophic level. Other potential examples of this three-way interaction could include mycophagous insects being consumed by insectivorous mammals such as antechinus or bandicoots, which are then in turn consumed by a predator like a quoll. However, there is no published evidence of fungal spores passing through three trophic levels.

There are many other predators in eastern Australia that may be dispersing fungi secondarily. Snakes and monitor lizards are likely to be particularly effective given that they generally consume their prey whole, meaning the incidental ingestion of fungal spores is highly probable (Elliott et al. 2019a). However, the effect of very lengthy digestion times on spore viability is unknown. Predatory birds like raptors and owls are also probable secondary dispersers of spores, either through scats or regurgitated pellets after consumption (Elliott et al. 2019b). Many raptors gut their prey leaving the gastrointestinal tract (Forsman et al. 2004) but will often still fly some distance to a tree or nest before disembowelling their prey. If these remains are then consumed by a scavenger, further dispersal of spores could occur. Red foxes are also likely to play a role in long-distance spore dispersal, given their predation on small native mycophagous mammals and evidence of secondary fungal dispersal from North America (Stephens et al. 2025). Feral cats (*Felis catus*) also predate small native mycophagous mammals, but may play less of a role as secondary fungal dispersers as they sometimes disregard unpalatable parts of their prey, such as the digestive tract, when not nutritionally stressed (Fisher et al. 2015).

### Viability of spores after secondary dispersal

A major assumption we make in this study is that mycorrhizal fungal spores remain viable after secondary dispersal, given that the spores have passed through (at least partially) the digestive tracts of two animals. Freezing and alcohol emersion of samples prevented a thorough assessment of spore viability in this study, but the majority of fungal spores we examined showed no evidence of any degradation of spore walls or ornamentation, suggesting they were still viable after secondary consumption by quolls. Other studies of mammalian predators secondarily consuming fungi have also found no evidence to suggest reduced spore viability after secondary dispersal (Elliott et al. 2024; Stephens et al. 2025). Previous studies on spore dispersal have shown that spores are still viable after ingestion and defecation by at least 40 species of primary mycophagists (Ori et al. 2021; Piattoni et al. 2014; Elliott et al. 2022), and several have shown that digestion by a mycophagist increases the likelihood of mycorrhiza formation (Claridge et al. 1992; Lamont et al. 1985; Reddell et al. 1997). Some species with longer gut retention times have been shown to be more effective at freeing spores from the asci and degrading spore walls, potentially increasing the chances of germination and mycorrhiza formation (Ori et al. 2021). The Pampas fox (*Lycalopex gymnocercus*), a similarly sized predator to quolls and Tasmanian devils, has been shown to disperse viable mycorrhizal fungi spores in its scats (Aguirre et al. 2021).

Parallels can also be drawn with seed dispersal, where germination success can be increased or decreased depending on the length of gut retention time and thickness of the seed coating (Nogales et al. 2015; Traveset et al. 2007). However, the majority of studies on the secondary dispersal of seeds found that they increased germination success relative to primary dispersers, rather than reduced it (Hämäläinen et al. 2017). Given this evidence, and the hardiness that allows fungal spores to remain viable in the environment over long periods of time (Nguyen et al. 2012), we suggest that spores are still viable after dispersal by secondary consumers. However, further research on the viability of fungal spores after secondary dispersal is necessary. If spore dispersal after passage through three or more trophic levels is investigated, further research on the viability of fungal spores would also be necessary.

### Conservation implications

A diverse community of fungal dispersers is important to forest ecosystems, as each species inhabits a slightly different microhabitat and exhibits behavioural differences that affect the location of fungal spore deposition, thus facilitating a greater dispersal capacity (Vernes et al. 2015). Unlike many of the smaller primary mycophagists that quolls and Tasmanian devils consume, both predators range widely over degraded habitat and between forest fragments (Andersen et al. 2017b; Hamer et al. 2021; Henderson et al. 2023). Dispersal of mycorrhizal fungal spores in these areas assists with fungal gene flow between fragments, recolonisation of vegetation and the development of seedling corridors (Bent et al. 2011; Claridge et al. 2001; Frank et al. 2009; Torrez et al. 2016). In this study, quolls at the Hebden site are likely to be important long-distance dispersers of mycorrhizal fungi into disturbed or fragmented landscapes, given they inhabit these areas of the site (Henderson et al. 2023). Both quolls and Tasmanian devils are likely to move fungal spores across ecotones and may assist in shifting vegetation communities by introducing specific mycorrhizal fungi, such as rainforest associated species, into new habitats like sclerophyll forest (Vernes and Dunn 2009).

The decline or extinction of fungal dispersers may drastically affect mycorrhizal fungal diversity in soils and thus negatively affect regeneration of associated plant species and forest resilience after disturbance (Dundas et al. 2018; Elliott et al. 2022; Liang et al. 2020). As the two largest extant marsupial carnivores in Australia (Menkhorst and Knight 2011), quolls and Tasmanian devils are likely responsible for many LDD events in habitats where they occur. Unfortunately, both species have declined across their range, particularly the Tasmanian devil, which has potentially reduced the number of LDD events that are occurring. As a result, the ability of fungal communities and their associated plant species to colonise new habitats, recolonise disturbed habitats or respond to climactic shifts may be diminished (Van Nuland et al. 2024; Vernes and Dunn 2009). The recognition of quolls and Tasmanian devils as long-distance dispersers of mycorrhizal fungi provides additional evidence to their importance to Australian forested ecosystems and highlights the need for the conservation of these two species and other native predators. Management actions such as reduced habitat clearing, increasing habitat connectivity and introduced predator control would benefit both quolls and Tasmanian devils (Jones et al. 2003), as well as plant and fungal communities more broadly.

## Conclusion

This study is the first to identify spotted-tailed quolls and Tasmanian devils as secondary consumers and dispersers of mycorrhizal fungi, an ecological role that has only recently been recognised among predators, but is likely widespread across insectivores, predators and scavengers. The high frequency of occurrence of fungi in scats and wide diversity of fungi that quolls consumed suggests that they are playing an important role in the LDD of mycorrhizal fungi in south-eastern Australia. Given limited sampling in this study, further investigation into Tasmanian devils as secondary consumers of mycorrhizal fungi should be conducted to clarify their role as long-distance fungal dispersers. Conservation of both quolls and Tasmanian devils may be beneficial to maintaining diverse fungal and plant communities, given their role as long-distance dispersers of mycorrhizal fungi. Greater research effort on the interrelationships between fungi, mycophagists and secondary consumers would further our knowledge of their respective roles in maintaining healthy ecosystems. Determining the viability of fungal spores after dispersal and whether this varies between species or trophic level would also allow a more complete understanding of the relative importance of different species to mycorrhizal fungi dispersal. It is also a reminder that more broadly there are still major gaps in our current understanding of predator ecology and ecosystem dynamics. 

## Data Availability

Data is available from the authors on reasonable request.
